# Intrathecal Injection of Mesenchymal Stromal Cell Cultured on 3D Fiber Ameliorates Multiple Organ Damage in Murine Lupus

**DOI:** 10.1093/stcltm/szac021

**Published:** 2022-04-25

**Authors:** Yuki Saito, Maki Miyajima, Sena Yamamoto, Norihiro Miura, Tsukasa Sato, Arisa Kita, Shogo Ijima, Mineko Fujimiya, Takako S Chikenji

**Affiliations:** Department of Anatomy, Sapporo Medical University School of Medicine, Sapporo, Japan; Graduate School of Health Sciences, Hokkaido University, Sapporo, Japan; Graduate School of Health Sciences, Hokkaido University, Sapporo, Japan; Graduate School of Health Sciences, Hokkaido University, Sapporo, Japan; Graduate School of Health Sciences, Hokkaido University, Sapporo, Japan; Department of Plastic and Reconstructive Surgery, Sapporo Medical University, Sapporo, Japan; Department of Oral Surgery, Sapporo Medical University, Sapporo, Japan; Department of Anatomy, Sapporo Medical University School of Medicine, Sapporo, Japan; Department of Anatomy, Sapporo Medical University School of Medicine, Sapporo, Japan; Graduate School of Health Sciences, Hokkaido University, Sapporo, Japan

**Keywords:** systemic lupus erythematosus, bone marrow, mesenchymal stromal cell, multiple organ damage, sympathetic nervous system

## Abstract

Up to 60% of patients with systemic lupus erythematosus (SLE) experience autonomic symptom. Sympathetic nervous system damage can cause dysfunction of the bone marrow that activates inflammatory cells, potentially causing multiple organ damage. We hypothesized that sympathetic nervous system damage would induce bone marrow dysfunction with multiple organ damage in SLE, and that multiple organ damage could be improved by therapy targeting the nervous system. Here, we showed that damage to autonomic nerves and Schwann cells occurred in the bone marrow and central nervous system of SLE model mice. A neurotoxic drug increased mortality and induced severe neuropathy and multiple organ damage, while a neuroprotective drug prevented multiple organ damage. The administration of bone marrow-derived mesenchymal stromal cells (BMSCs) cultured on a 3-dimensional fiber scaffold improved bone marrow neuropathy, skin lesions, kidney function, and mortality. Our results reveal that bone marrow neuropathy influence multiple organ damage associated with SLE, and improvement of bone marrow neuropathy by intrathecal injection of BMSC may be a target for SLE multiple-organ damage.

## Graphical Abstract

**Graphical Abstract:**
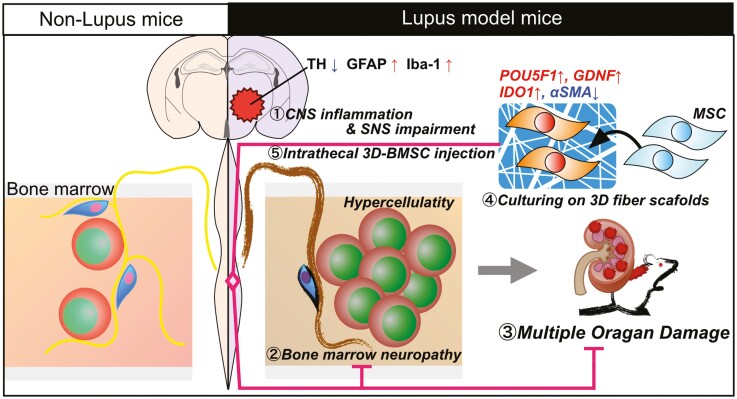

**Significance Statement**

Our study highlights the roles of bone marrow neuropathy and multiple organ damage in a murine lupus model. The neuroprotective effects of intrathecal bone marrow mesenchymal stromal cells (BMSC) injection may serve as a therapeutic option for restoring bone marrow and alleviating multiple organ damage. In addition, BMSCs had a decreased therapeutic effect when derived from patients with systemic lupus erythematosus; therefore, we propose pre-conditioning these cells by culturing them on 3D fiber scaffolds to improve the benefits of autologous BMSC therapy.

## Introduction

Systemic lupus erythematosus (SLE) is an incurable autoimmune disease characterized by hyperactive immune cells, serum autoantibodies, and damage to multiple organs including the kidney, skin, vasculature, and brain.^1^ While the exact pathogenic mechanism of SLE is still unknown, it is apparent that genetic, environmental, hormonal, and epigenetic factors result in the generation of autoantibodies, immune complexes, and inflammatory cytokines that can damage multiple organs.^1^

Neuropsychiatric manifestations are among the most common in SLE targeting the central, peripheral, and autonomic nervous system. Up to 75% of patients experience central nervous system (CNS) involvement, and 60% of patients with SLE experience autonomic symptoms.^2-4^ CNS inflammation caused by chronic systemic inflammation exacerbates sympathetic nerve damage.^5,6^ The sympathetic nervous system (SNS) is instrumental for orchestrating inflammatory cell release from the bone marrow, which directs sympathetic innervation organ,^7^ and the SNS also supports the bone marrow microenvironment as a hematopoietic stem cell (HSC) niche.^8^ Glial fibrillary acid protein (GFAP)-positive non-myelinating Schwann cells are key elements of the HSC niche. SNS and Schwan cell damage in bone marrow, called bone marrow neuropathy, causes cytokine storms, resulting in bone marrow fibrosis and osteosclerosis in myeloproliferative neoplasms.^9^ In patients with SLE, bone marrow abnormalities such as bone marrow fibrosis, myelodysplastic syndromes, and hypercellularity have been reported,^10-12^ and bone marrow transplantation improves lupus nephropathy in patients with SLE and MRL/*lpr* mice.^13,14^ Another report suggested that an impaired bone marrow microenvironment causes bone marrow-derived cells to damage tissues by migrating to target organs and expressing cytokines.^15^

These studies suggest that dysfunction of the SNS in SLE can cause bone marrow abnormalities with the potential to drive multiple organ damage through dysregulation of inflammatory cell release from the bone marrow. We hypothesized that SNS dysfunction causes damage to multiple organs and tissues, including the bone marrow HSC niche in SLE, and that this damage could be improved by therapy targeting the nervous system.

## Materials and Methods

### Mice

The Committee of the Animal Experimentation Center of the Sapporo Medical University School of Medicine approved all animal protocols (#17-080). Mice were maintained in an enclosed, pathogen-free facility in a 12-hour light and dark cycle. Female MRL/*lpr* mice were used as an SLE mouse model, and haplotype-matched female MRL/*Mpj* mice were used as phenotypic controls (Sankyo Lab Service, Tokyo, Japan). Mice were euthanized by an overdose of isoflurane at 12-28 weeks of age, and tissue samples were harvested. Mice that lost more than 30% of body weight relative to controls, or exhibited persistent crouching or lying down, were also euthanized as humane endpoint.

### Pharmacological Treatments

Twelve-week-old MRL/lpr mice were randomized for pharmacological treatment (*n* = 36). The neuroprotective drug 4-methylcatechol (4MC; Sigma-Aldrich, St. Louis, MO, USA) was administered at 10 µg/kg through intraperitoneal (i.p.) injection 5 times a week for 12 weeks (*n* = 12). The sympathetic neurotoxic drug 6-hydroxydopamine (6OHDA; Sigma-Aldrich) was administered at 100 µg/g dissolved in 0.01% ascorbic acid in saline through i.p. injection, and 3 days after the first injection, 250 µg/g of 6OHDA was administrated through i.p. injection (*n* = 12). Saline was administered through i.p. injection as vehicle control (*n* = 12).

### Cell Culture and Treatments

Experiments using human samples were approved by the institutional review board at the Sapporo Medical University (Approval Number: #302-1134), and all experiments were performed in accordance with relevant guidelines and regulations. Heparinized bone marrow (10 mL) was aspirated from the iliac crest during total hip arthroplasty procedures from patients with a history of SLE, and without history of autoimmune diseases. The aspirated sample was centrifuged at 300 g for 10 minutes. The cell fraction was isolated and suspended in complete medium (Dulbecco’s modified Eagle’s medium [DMEM; Sigma-Aldrich] containing 4500 mg/L glucose, 10% fetal bovine serum [FBS], and 1% penicillin-streptomycin) equal in volume to the originally aspirated bone marrow. Suspended bone marrow cells (1 mL) were plated in a 100-mm polystyrene culture dish in 9 mL of complete medium. Cultures were maintained at 37 °C in a humidified atmosphere containing 5% CO_2_. Nonadherent cells were removed after 1 week of adhesion, and the culture medium was replaced twice a week. Bone marrow-derived mesenchymal stromal cells (BMSCs) were harvested as passage 0 after 3-4 weeks of culture. After trypsinization, 4 × 10^5^ BMSCs were seeded in 100-mm polystyrene culture dishes in 9 mL of complete medium (passage 1). At passage 2, BMSCs were cultured on polystyrene culture dishes (BMSCs) or on 3-dimensional culture membranes (VECELL; Vessel. Fukuoka, Japan) (3D-BMSCs). After 48-hour culture, detached cells were used for cell treatments. For the experiment of in vivo tracking of BMSCs, cells were labeled with PKH26 according to the manufacturer’s instructions (Sigma-Aldrich).

Eighteen-week-old MRL/*lpr* mice were randomized for cell treatment. For intralumbar injection, 2.5 × 10^4^ MSCs were suspended in 5 µL of phosphate-buffered saline (PBS) and injected into the lumbar subarachnoid space. Briefly, mice were placed in the Trendelenburg position (30 °C), the vertebrae at L4 and L5 were exposed, and a 30-gauge needle was inserted between these vertebrae. The BMSCs were injected when the tail-flick reflex was observed. The needle was maintained in the lumbar subarachnoid space for 1 minute after injection to minimize the leakage of BMSCs. PBS injection was conducted with the same methods for vehicle control.

### Proteinuria

Proteinuria was assessed and scored semiquantitatively with Albustix test strips (Siemens Healthineers, Malvern, PA). Scores were as follows: grade 0, 0 mg/dL; grade ±, <30 mg/dL; grade 1+, ≥30 mg/dL; grade 2+, ≥100 mg/dL; grade 3+, ≥300 mg/mL; and grade 4+, ≥1000 mg/dL.

### Skin Lesions

Skin lesions were scored on a scale of 0-3, as follows: snout skin, score 0 for no lesion, 0.5 for minor lesion (<50% involvement), and 1 for major lesion (≥50% involvement); back skin, score 0 for no lesion, 0.5 for minor lesion (<φ10-mm lesion), 1 for major lesion (≥φ10-mm lesion), and 1.5 for major lesion with scab formation; and eye skin, score 0 for no lesion and 0.5 for any lesion.

### Histological Staining and Imaging

Bone and kidney were harvested and immersed in 4% paraformaldehyde for 24 h at 4 °C, then paraffin-embedded tissue blocks were made and sectioned at 5 µm. Sections were deparaffinized and hydrated using an ethanol gradient. Bone sections were stained with hematoxylin and eosin (H&E), and kidney sections were stained with periodic acid-Schiff (PAS). For immunohistochemistry of bone marrow, antigen retrieval was performed with EDTA pH 9 or HistoVT One (Nacalai Tesque, Kyoto, Japan). After washing 3 times with PBS, the sections were incubated in 0.01 M PBS containing 0.3% Triton-X (PBS-T) and treated with 2% bovine serum albumin (BSA) for 60 minutes at room temperature (RT). After washing with 0.01 M PBS-T, the sections were incubated with Alexa Fluor 594-conjugated anti-GFAP antibody (1:100; 644708; Biolegend, San Diego, CA, USA), and nuclei were stained using 4ʹ,6-diamidino-2-phenylindole (DAPI) (1:1000; Dojindo, Kumamoto, Japan). After washing, tissue sections were mounted with Vectashield (Vector Laboratories, Burlingame, CA, USA). Sections were observed with confocal laser scanning microscopy (Nikon/A1; Nikon, Tokyo, Japan) and fluorescence microscopy (BZ-X700; Keyence, Osaka, Japan).

Brain samples were fixed in 4% paraformaldehyde overnight. The following day, the tissues were transferred to 20% sucrose in phosphate buffer and incubated overnight, frozen in OCT compound using liquid nitrogen, and stored at −80 °C until use. Cryosections (8-µm thick) were prepared using a cryostat. The sections were incubated in 0.01 M PBS containing 0.3% Triton-X (PBS-T) and treated with 2% BSA for 60 minutes at RT. After washing with 0.01 M PBS-T, the sections were incubated with primary antibodies at 4 °C overnight, followed by secondary staining. Alexa Fluor 594-conjugated anti-GFAP antibody (1:100; 644708; Biolegend), anti-tyrosine 3 hydroxylase antibody (1:100; E-AB-33093; Elabscience, Houston, TX, USA), and anti-Iba-1 antibody (1:400; 019-19741; Wako, Osaka, Japan) were used as primary antibodies. For secondary antibodies, we used Alexa Fluor 488-conjugated IgG (1:400; Jackson ImmunoResearch, West Grove, PA, USA), and nuclei were stained using DAPI (1:1000; Dojindo). After washing, tissue sections were mounted with Vectashield (Vector Laboratories). Sections were observed with a confocal laser scanning microscope (Nikon/A1; Nikon) and fluorescence microscopy (Axio Observer7 (ZEISS) and BZ-X700; Keyence).

### Histopathological Evaluation of the Kidney

PAS-stained paraffin sections were used for morphological evaluation, and each glomerulus was graded on a scale of 0-3 as previously described by Ikeda et al: grade 0, no lesions in the glomerulus; grade 1, mild cell proliferation and/or cell infiltration; grade 2, the same as grade 1 with mesangial proliferation, lobulation, and hyaline droplets; and grade 3, the same as grade 2 with crescent and granuloma formation and/or hyalinosis.^16^ The histopathological score in each mouse was calculated as the mean value of the grades of 20 glomeruli.

### RNA Extraction and Quantitative Real-Time PCR

Total RNA was isolated from bone marrow using Tri Reagent (Molecular Research Center, Cincinnati, OH, USA), and the RNA was reverse transcribed into cDNA using an Omniscript RT Kit (205113; Qiagen, Hilden, Germany) or iScript cDNA Synthesis Kit (1708891; Bio-Rad, Hercules, CA, USA). Quantitative PCR was performed using one of 2 methods: with Power SYBR Green Master Mix (4368702; Applied Biosystems, Foster City, CA, USA) using the Applied Biosystems 7500 Real-Time PCR System (Applied Biosystems) under the following cycling conditions: 50 °C for 2 minutes and 95 °C for 10 minutes followed by 40 cycles of amplification (95 °C for 15 s and 60 °C for 1 minutes); or with SsoAdvanced Universal SYBR Green Supermix (172-5270; Bio-Rad) using the QuantStudio 3 Real-Time PCR System (Applied Biosystems) under the following cycling conditions: 95 °C for 20 s followed by 40 cycles of amplification (95 °C for 15 s and 60 °C for 1 minute). Expression levels were normalized against the corresponding levels of *Gapdh* and *Actb* mRNA. Specific primer sequences used for PCR are listed in Table 1. The ΔΔ*C*_t_ method was used to compare data.

### Coculture of MSC and TGW

Three-dimensional culture membranes (VECELL) were used for transwell coculture. First, 2.0 × 10^4^ BMSCs were plated on the VECELL inserts and cultured in DMEM with 10% FBS for 48 hours. Next, 4.0 × 10^4^ TGW cells were grown independently from BMSCs in minimum essential medium Eagle with Earle’s salts (MEM: Sigma-Aldrich) with 10% FBS for 48 hours. VECELL inserts themselves with BMSCs were transferred into the TGW-cultured plates, and the medium was replaced with fresh DMEM with 10% FBS. Cells were treated with or without recombinant GDNF protein (50 ng/mL; 700401; Biolegend) and GDNF-neutralizing antibody (1 µg/mL; AF-212-NA; R&D Systems, Minneapolis, MN, USA) for 48 hours. To prepare conditioned media (CM) from 2D-BMSC and 3D-BMSC, the cells were washed with PBS and cultured with DMEM including 10% FBS. Twenty-four hours later, CM were collected. The CM collected from 2D-BMSC and 3D-BMSC were transferred into TGW cells and cultured for 48 hours. After 48 hours coculture or cultured in CM, TGW cells were fixed with 4% paraformaldehyde for 15 minutes at RT and immunostained with TH antibody (1:100; AB152; Merck, Darmstadt, Germany). Briefly, fixed cells were washed with PBS, then incubated in PBS-T for 15 minutes and treated with 2% BSA for 60 minutes at RT. After washing with PBS-T, the cells were incubated with primary antibodies at 4 °C overnight, followed by secondary staining with Cy3-conjugated IgG (1:400; Jackson ImmunoResearch) and nuclear staining with DAPI (1:1000). Cells were observed with a BZ-X700 fluorescence microscope (Keyence). To quantify TH expression, fluorescence intensity was measured using ImageJ (National Institutes of Health). TGW cells (Cell No. JCRB0618) were provided by the Japanese Collection of Research Bioresources Cell Bank (Osaka, Japan).^17^

### Characterization of 2D-BMSC and 3D-BMSC

2D-BMSC and 3D-BMSC were cultured on 8-well chamber slides or on Vecell for osteogenic and adipogenic differentiation, and detached 2D-BMSC and 3D-BMSC were cultured in 15-mL conical tubes for chondrogenic differentiation. Mesenchymal stem cell functional identification kits (R&D Systems) were used to confirm the differentiation potential. Immunofluorescence staining with anti-osteocalcin, anti-FABP4, and anti-aggrecan antibodies was used for detecting the osteogenic, adipogenic, and chondrogenic differentiation, respectively, according to the manufacturer’s instructions.

2D-BMSC and 3D-BMSC were cultured on 8-well chamber slides or on Vecell were immunostained for Surface maker expression analysis. Cells were fixed in 4% paraformaldehyde for 15 minutes at RT. Fixed cells were incubated in 0.01 M PBS containing 0.3% Triton-X (PBS-T) and treated with 2% BSA for 60 minutes at RT. After washing with 0.01 M PBS-T, the sections were incubated with primary antibodies at 4 °C overnight, followed by secondary staining. Anti-CD90 antibody (1:100; ab14450; Abcam, Cambridge, UK), anti-CD73 antibody (1:100: ab175396; Abcam), anti-CD45 antibody (1:1000: ab8216; Abcam), and anti-CD34 antibody (1:250: ab81289; Abcam) were used as primary antibodies. For secondary antibodies, we used Alexa Fluor Plus 555-conjugated anti-mouse or rabbit IgG (1:400; Jackson ImmunoResearch, West Grove, PA, USA), and nuclei were stained using DAPI (1:1000; Dojindo). Cytoskeleton was stained with Phalloidin-iFluor 647 Reagent (ab176759; Abcam) according to the manufacturer’s instructions. After washing, cells were mounted with Vectashield (Vector Laboratories). Cells were observed with fluorescence microscopy (Axio Observer7 (ZEISS)). Cell size and aspect ratio were measured using ImageJ (National Institutes of Health). Briefly, the cell body was outlined using the drawing/selection tools, and the area was measured using the analyze tool as cells size. The cell aspect ratio was calculated with the major axis divided by the minor axis (Islam 2016).

### Behavioral Analysis

The object recognition test was used as a recognition memory test. During the first session, mice were presented with 2 identical objects in a 30 cm × 30 cm × 30 cm box. During the second session, conducted 1 hour after the first session, one of the 2 objects was replaced by a new object, and the time spent exploring the new object was used as an index of recognition memory. To evaluate anxiety-like behavior, we used the elevated plus-maze test. We measured the time spent in the open and closed arms during 5 minutes of free exploration. The tail suspension test was performed to assess depression-like behaviors. Mice were suspended by their tails with tape for 6 minutes, and the immobile time was measured as an index of depression-like behavior. All behavioral tests were recorded and analyzed with a video tracking system (ANY-maze; Muromachi Kikai, Tokyo, Japan).

### Statistical Analysis

Quantitative data are shown as means and standard errors in dot plots using ggplot2, which is a plotting system for R based on The Grammar of Graphics (The R Foundation for Statistical Computing, Vienna, Austria). Normality was assessed using the Shapiro-Wilk test. The pairwise t-test was used for comparison between 2 groups, and a one-way analysis of variance (ANOVA) or the Kruskal-Wallis test was conducted to assess differences among 3 groups or more. Pairwise comparisons were made only when the one-way ANOVA or Kruskal-Wallis test indicated statistical significance. P-values for multiple comparisons were adjusted by the Holm method. Statistical analyses were performed using EZR, which is a graphical user interface for R.^18^ Two-sided *P*-values <.05 were considered statistically significant.

## Results

### MRL/*lpr* Mice Exhibit Central Nervous System Impairment

**Figure 1.**
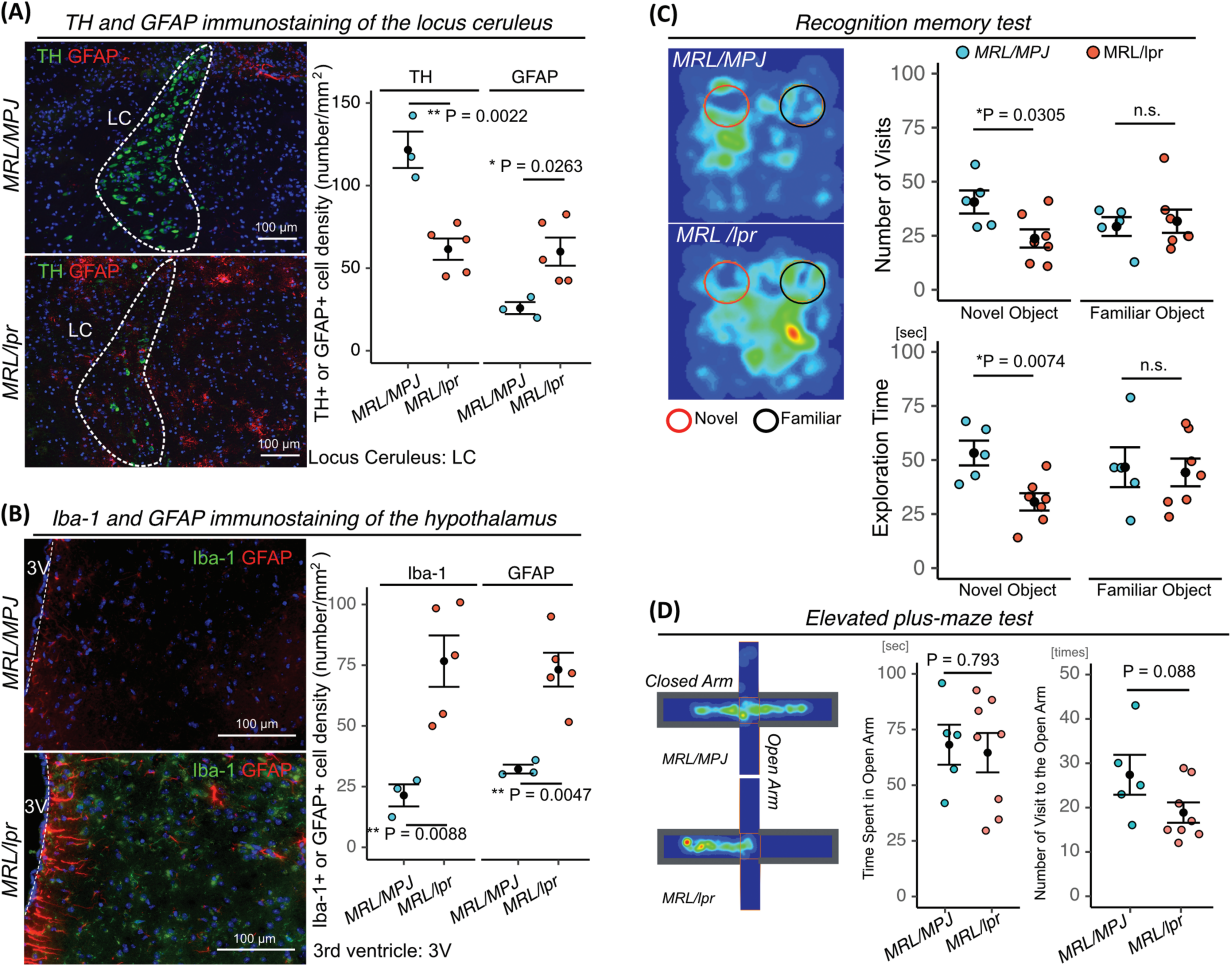
MRL/*lpr mice* demonstrate CNS impairment. (**A**) Representative images of TH and GFAP immunostaining of the locus ceruleus in MRL/*MPJ* (*n* = 3) and MRL/*lpr* mice (*n* = 5), with quantifications. (**B**) Representative images of Iba-1 and GFAP immunostaining of the hypothalamus in MRL/*MPJ* (*n* = 3) and MRL/*lpr* mice (*n* = 5), with quantifications. (**C** and **D**) Cumulative heat maps of time spent during the object recognition test and elevated plus-maze. A longer time spent is depicted in red and no time spent is depicted in black. (C and D) Quantitation of the number of visits to novel and familiar objects or open arms and the time spent exploring each or open arm in MRL/*MPJ* (*n* = 5) and MRL/*lpr* mice (*n* = 7). Quantitative data are shown as means ± SEs in dot plots. *P*-values were determined by 2-tailed Student’s *t* test (*P < 0.05, **P < .001).

To determine whether lupus-prone mice exhibited impairment of the central or autonomic nervous system, we used 18-week-old MRL/*lpr* mice as an SLE model and MRL/*MPJ* mice as controls. First, we confirmed the onset of SLE symptoms in MRL/*lpr* mice by evaluating dsDNA and BAFF, kidney histopathology, and proteinuria (Supplementary Fig. S1). Both dsDNA and BAFF are markers of SLE disease activity.^19,20^ To determine whether the SNS was impaired in MRL/*lpr* mice, we measured the expression of tyrosine hydroxylase (TH) and GFAP, an astrocyte marker, in the locus ceruleus, the major source of noradrenaline.^21^ Expression of TH was significantly lower in MRL/*lpr* mice than in MRL/*MPJ* mice, whereas expression of GFAP was significantly higher (Fig. 1A). We next evaluated CNS inflammation in the hypothalamus, a region that plays a major role in central control of the autonomic nervous system. Expression levels of ionized calcium-binding adaptor molecule 1 (Iba1), a microglia marker, and GFAP were significantly higher in MRL/*lpr* mice than in MRL/*MPJ* (Fig. 1B). We subsequently performed behavioral tests to investigate the presence of CNS lupus. MRL/*lpr* mice exhibited cognitive defects in a recognition memory test, but no anxiety-like phenotype in the elevated plus-maze test (Fig. 1C and D). From these results, we confirmed that MRL/*lpr* mice exhibited autonomic nervous impairment with the inflammation in locus ceruleus and hypothalamus.

### MRL/*lpr* Mice Exhibit Bone Marrow Neuropathy

**Figure 2.**
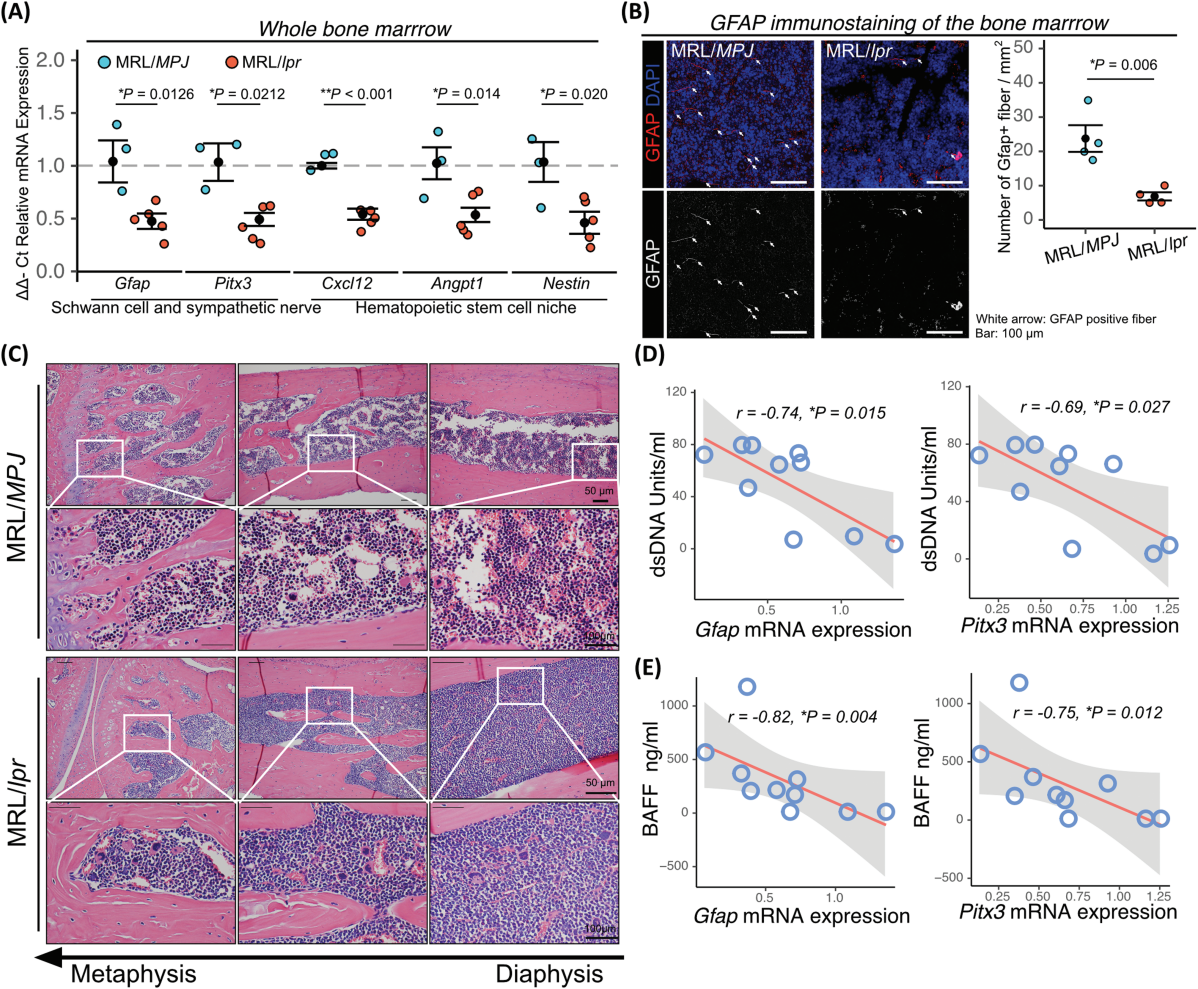
MRL/*lpr* mice exhibit bone marrow neuropathy. (**A**) Relative mRNA expression in bone marrow from MRL/*MPJ* (*n* = 3) and MRL/*lpr* mice (*n* = 5). (**B**) Representative images of GFAP immunostaining of MRL/*MPJ* (*n* = 4) and MRL/*lpr* bone marrow (*n* = 4), and quantifications. Arrow indicates GFAP fiber. (**C**) Representative images of H&E staining of MRL/*MPJ* and MRL/*lpr* bone marrow. (**D** and **E**) Correlation of *Gfap* and *Pitx3* mRNA expression with dsDNA and BAFF in MRL/*MPJ* and MRL/*lpr* mice. Correlations were examined statistically using Pearson’s correlation coefficient, and 95% confidence intervals are shown with gray fill. Quantitative data are shown as means ± SEs in dot plots. *P*-values were determined by 2-tailed Student’s *t* test (*P < .05, **P < .001).

Next, we assessed the presence of bone marrow neuropathy. MRL/*lpr* bone marrow exhibited significant reductions in expression of *Gfap* and *Pitx3* (Fig. 2A), the latter of which encodes a transcription factor in dopamine neurons,^22,23^ as well as in the number of GFAP-positive fibers relative to MRL/*MPJ* (Fig. 2B). MRL/*lpr* also exhibited significantly reduced the expression of *Cxcl12*, *Angpt1*, and *Nestin* (Fig. 2A), an HSC supportive factor.^24^ MRL/*lpr* also exhibited hypercellularity in bone marrow (Fig. 2C). Furthermore, the expression of *Gfap* and *Pitx3* in bone marrow was correlated with the level of dsDNA and BAFF (Fig. 2D and E). Based on the above results, we hypothesized that bone marrow neuropathy is associated with SLE symptoms.

### The Neuroprotective Effect of 4MC Treatment Prevents Bone Marrow Impairments and Nephropathy in MRL/*lpr* Mice

**Figure 3.**
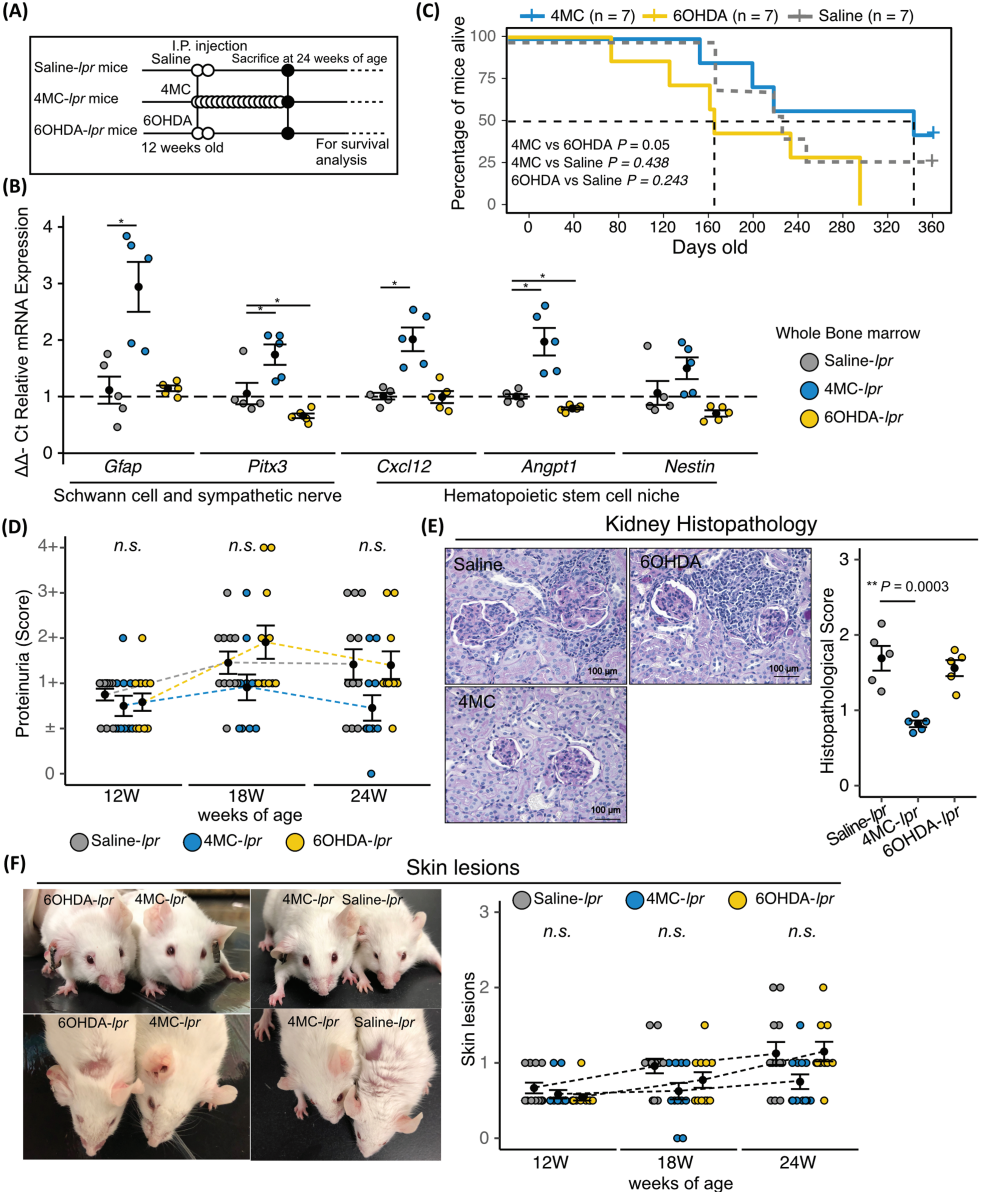
Neuroprotective effects of 4MC treatment prevent aggravation of lupus symptoms in *MRL/lpr* mice. (**A**) Schematic diagram of procedures for 4MC and 6OHDA treatment of MRL/*lpr* mice. (**B**) Relative mRNA expression of genes related to Schwann cells, sympathetic nerves, and the hematopoietic stem cell niche in the bone marrow (*n* = 5 in each group). (**C**) Kaplan-Meier survival curves for saline-, 4MC-, and 6OHDA-treated mice (*n* = 7 in each group). (**D**) Semi-quantitative data of proteinuria after 4MC and 6OHDA treatment (*n* = 12 in each group). (**E**) Representative images of PAS-stained kidneys from saline-, 4MC-, and 6OHDA-treated mice, and quantitations of the histopathological scores (*n* = 5 in each group). (**F**) Representative images of skin lesions in saline-, 4MC-, and 6OHDA-treated mice, and quantitations (*n* = 12 in each group). Quantitative data are shown as means ± SEs in dot plots. *P*-values were determined by one-way ANOVA adjusted by the Dunnett method or Steel’s multiple comparison-Wallis (*P < .05, **P < .001).

To determine whether bone marrow neuropathy affects the severity of SLE symptoms, we examined the effects of the neuroprotective drug 4-methylcatechol (4MC) and the sympathetic neurotoxic drug 6-hydroxydopamine (6OHDA) in 12-week-old presymptomatic MRL/*lpr* mice (Fig. 3A). In bone marrow neuropathy studies, 4MC has been used for its neuroprotective effect, and 6OHDA has been used for SNS denervation.^9,25^ We confirmed that mRNA expression levels of *Gfap* and *Pitx3* were higher in 4MC-treated MRL/*lpr* mice (4MC-*lpr* mice) than in saline-treated MRL/*lpr* mice (Saline-*lpr* mice), and that mRNA expression was significantly lower in *Pitx3* in 6OHDA-treated MRL/*lpr* mice (6OHDA-lpr mice) than in Saline-*lpr* mice (Fig. 3B). Furthermore, *Angpt1* expression levels, which were reduced in the presence of bone marrow neuropathy,^9^ were also lower in 6OHDA-*lpr* mice (Fig. 3B). Next, we investigated mortality, severity of renal dysfunction, and skin lesions. Kaplan-Meier survival curves revealed that survival of 6OHDA-*lpr* mice was reduced (Fig. 3C). The proteinuria score did not differ significantly between 6OHDA-*lpr*, 4MC-*lpr*, and Saline-*lpr* mice, but 24-week-old 4MC-*lpr* mice exhibited improved glomerulonephritis and cell infiltration into the tubulointerstitial space than Saline-*lpr* mice (Fig. 3D and E). The skin lesion score did not differ significantly among the 3 types of mice (Fig. 3F). Next, we examined whether sympathetic neurotoxicity caused by 6OHDA administration at 6 weeks of age-induced multiple organ damage at 12 weeks of age, the time point at which no symptoms were previously observed; that is, we sought to determine whether multiple organ damage was accelerated. MRL/*lpr* mice that received 6OHDA at 6 weeks of age exhibited glomerulonephritis and cell infiltration into the tubulointerstitial space, whereas MRL/*lpr* that received no 6OHDA did not (Supplementary Fig. S2A and B). MRL/*lpr* mice that received 6OHDA had higher scores for proteinuria and skin lesions than MRL/*MPJ* mice (Supplementary Fig. S2C and D). MRL/*MPJ* mice treated with or without 6OHDA did not exhibit significant differences in kidney histopathology, proteinuria, or skin lesions (Supplementary Fig. S2A-D). From these experiments, bone marrow neuropathy can promote the progression of multiple-organ damage in lupus model mice; however, bone marrow neuropathy induction in non-lupus mice did not exhibit multiple-organ damage. Therefore, we hypothesis that treatment for bone marrow neuropathy could improve multiple-organ damage in lupus model mice.

### BMSCs Cultured on a 3-Dimensional Fiber Scaffold (3D-BMSCs) Markedly Improve Bone Marrow Neuropathy and SLE-Related Symptoms

**Figure 4.**
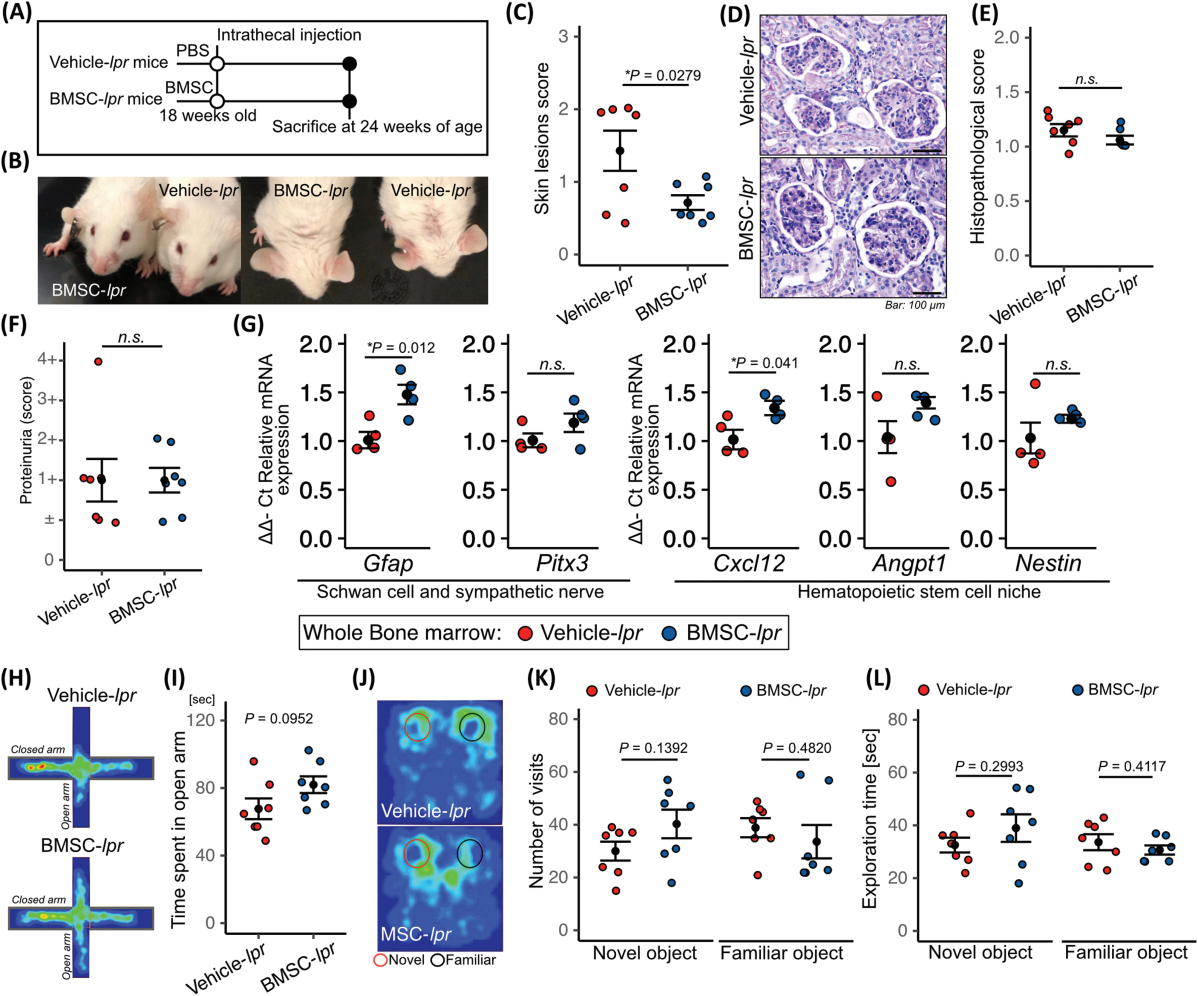
Intrathecal BMSC injection of bone marrow neuropathy into lupus-prone mice. (**A**) Schematic diagram of the procedure for intrathecal BMSC injection in MRL/*lpr* mice. (**B**) Relative mRNA expression of genes related to Schwann cells, sympathetic nerves, and the hematopoietic stem cell niche in the bone marrow after BMSC treatment. Representative images of skin lesions in vehicle- and BMSC-treated mice (**C**), and the quantitative data (**D**). Representative images of PAS staining of kidneys from vehicle- and BMSC-treated mice (**E**), and the quantitative data of the histopathological scores (**F**). (**G**) Semi-quantitative data of proteinuria after BMSC treatment. (**H**) Cumulative heat maps of the time spent in the elevated plus-maze. A longer time spent is depicted in red and no time spent is depicted in black. (**I**) Quantified data of the time spent in the open arm. (**J**) Cumulative heat maps of the time spent during the object recognition test. A longer time spent is depicted in red and no time spent is depicted in black. Quantified data of the number of visits to the novel and familiar objects (**K**) and the exploration times of each (**L**). Quantitative data for each specimen are shown as means ± SEs in dot plots. *P*-values were determined by 2-tailed Student’s *t* test (*P < .05, **P < .001).

**Figure 5.**
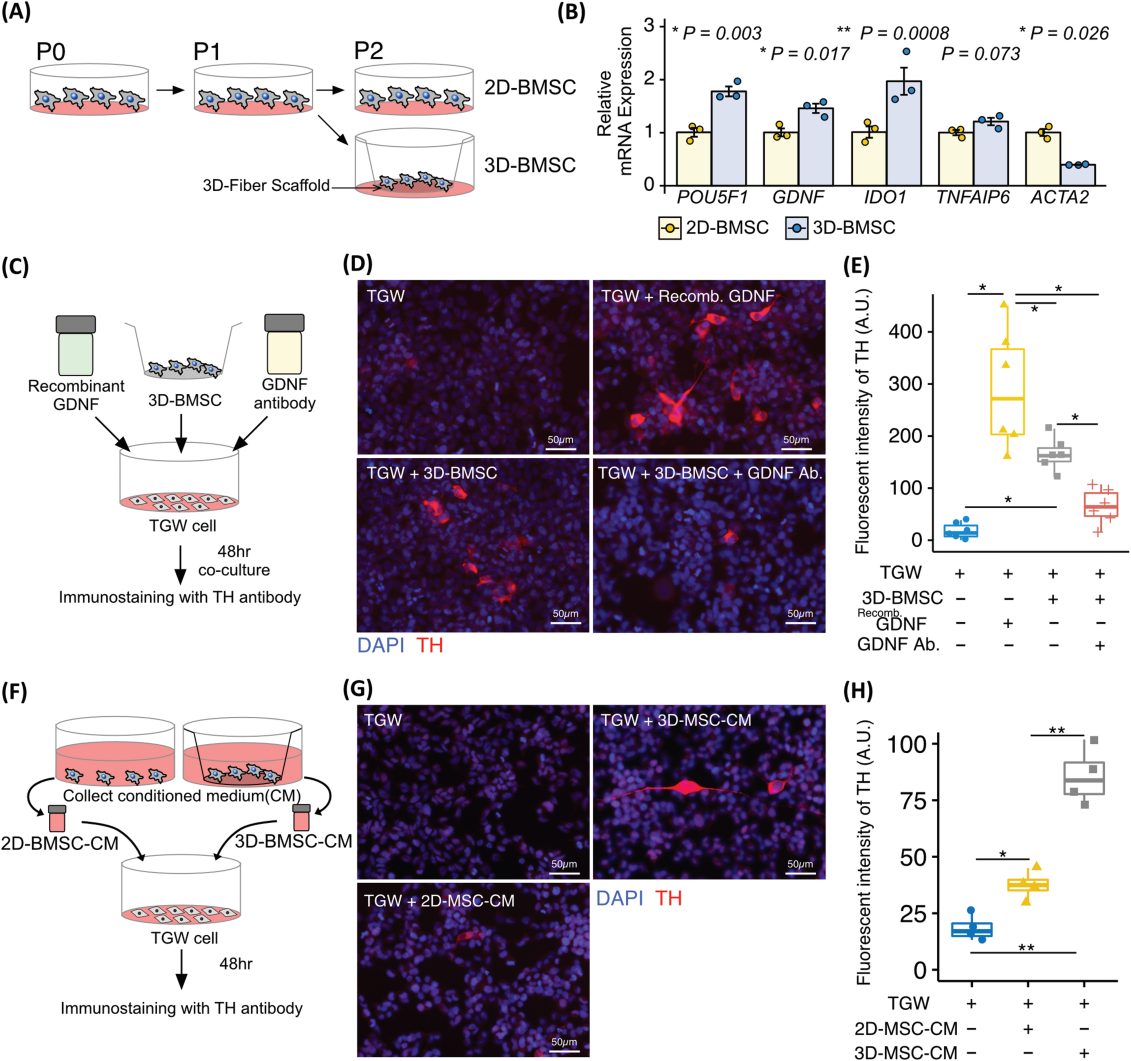
Characteristic changes of BMSCs cultured on a 3D fiber scaffold. (**A**) Schematic diagram of the cell preparation procedures. (**B**) Relative mRNA expression of *POU5F1*, *GDNF*, *IDO1*, *TNDAIP6*, and *ACTA2* in MSCs derived from patients with SLE and those cultured on a 3D fiber scaffold. (**C**) Schematic diagram of TGW cells cocultured in transwells with BMSCs. (**D**) Representative images of TH (red) and DAPI (blue) expression in TGW cells after 48-hours transwell coculture with or without BMSCs, recombinant GDNF, and anti-GDNF antibody, and (**E**) quantification of TH expression intensity. (**F**) Schematic diagram of TGW cells cultured with 2D-BMSC-derived conditioned media (2D-BMSC-CM) and 3D-BMSC-CM. (**G**) Representative images of TH (red) and DAPI (blue) expression in TGW cells after 48-h culture with 2D-BMSC-CM and 3D-BMSC-CM, and (E) quantification of TH expression intensity. Quantitative data are shown as means ± SEs or as medians with IQRs and 1.5 times the IQR, and are displayed in a dot plot or box-and-whisker plot. *P*-values were determined by 2-tailed Student’s *t* test or one-way ANOVA adjusted by the Holm method (*P < .05, **P < .001).

We next asked whether treatment for bone marrow neuropathy affect multiple-organ damage in lupus model mice. In this study we focused on MSC therapy because there is evidence that MSCs promote neuroregeneration.^26,27^ To achieve the improvement of bone marrow neuropathy, we performed lumbar intrathecal injection because the sympathetic innervation of the bone marrow is derived from thoracolumbar spinal cord.^28^ First, patients with SLE derived-BMSCs were injected into 18-week-old MRL/*lpr* mice (Fig. 4A). Intrathecal injection of BMSCs improved skin lesion but did not improved kidney histopathological score, and proteinuria (Fig. 4B-F). The mRNA expression of *Gfap* and *Cxcl12* were significantly increased in BMSC treated MRL/*lpr* mice (BMSC-*lpr*) (Fig. 4G), whereas the mRNA expression of *Pitx3*, *Angpt1*, and *Nestin* were not (Fig. 4G). BMSC-*lpr* did not improved cognitive defects in a recognition memory test and anxiety-like phenotype in the elevated plus-maze test (Fig. 4H-L). Since the bone marrow of patients with SLE is likely to be abnormal,^10^ we hypothesized that BMSCs derived from this source may have a diminished therapeutic effect. To solve this problem, we performed cell cultures using a 3D fiber scaffold. We confirmed both of the BMSCs cultured on a 2-dimensional plastic scaffold and 3D-BMSCs exhibits the property of BMSCs which expressed CD90, CD73 and negative for CD45, CD34 (Supplementary Fig. S3A and B). In addition, both of the BMSCs exhibits the trilineage mesenchymal differentiation to osteoblasts, adipocytes, and chondroblasts (Supplementary Fig. S3C). We also identified that 3D-BMSC exhibited equivalent cell proliferation with 2D-BMSC (Supplementary Fig. S3D), and 3D-BMSC exhibited spindle-shape and the cell size of 3D-BMSC is significantly smaller than that of 2D-BMSC (Supplementary Fig. S3E-G). We found that 3D-BMSCs significantly upregulated the mRNA expression of *POU5F1*, *IDO1*, and *GDNF*, and decreased that of *ACTA2*, compared with BMSCs cultured on a 2-dimensional plastic scaffold (2D-BMSC; Fig. 5A and 5B). Since TNF-α is known to be increased in serum and cerebrospinal fluid in patients with SLE and mice,^2,4,29^ we examined that the response of BMSC by TNF-α stimulation. We found that TNFα stimulated 3D-BMSC significantly increased the expression levels, 5.9-fold *GDNF*, 27.2-fold *IGF1*, 23.1-fold *TNFAIP6*, and 3,408-fold *IDO1* expression as compared with 2D-BMSC (Supplementary Fig. S3H). GDNF is known to promote the survival and morphological differentiation of dopaminergic neurons.^30,31^ To determine whether GDNF derived from 3D-BMSCs promotes the differentiation of dopaminergic neurons, we performed coculture experiments with 3D-BMSCs and TGW, a human neuroblastoma cell line^17^ (Fig. 5C). As shown previously, TGW cells express TH when stimulated by GDNF^32^; in this study, TGW cocultured with 3D-BMSCs promoted TH expression that was inhibited by GDNF-neutralizing antibodies (Fig. 5D, E). In addition, to test whether 3D-BMSC has strong effect to promote TH expression than that of 2D-BMSC, TGW cells were cultured with CM collected from 2D-BMSC and 3D-BMSC. We found 3D-MSC-derived CM significantly increased the TH expression in TGW cells than that of 2D-MSC derived CM (Fig. 5F-H). From these results, 3D-BMSC has the potential to improve the therapeutic efficacy of the multiple organ damage in murine lupus.

**Figure 6.**
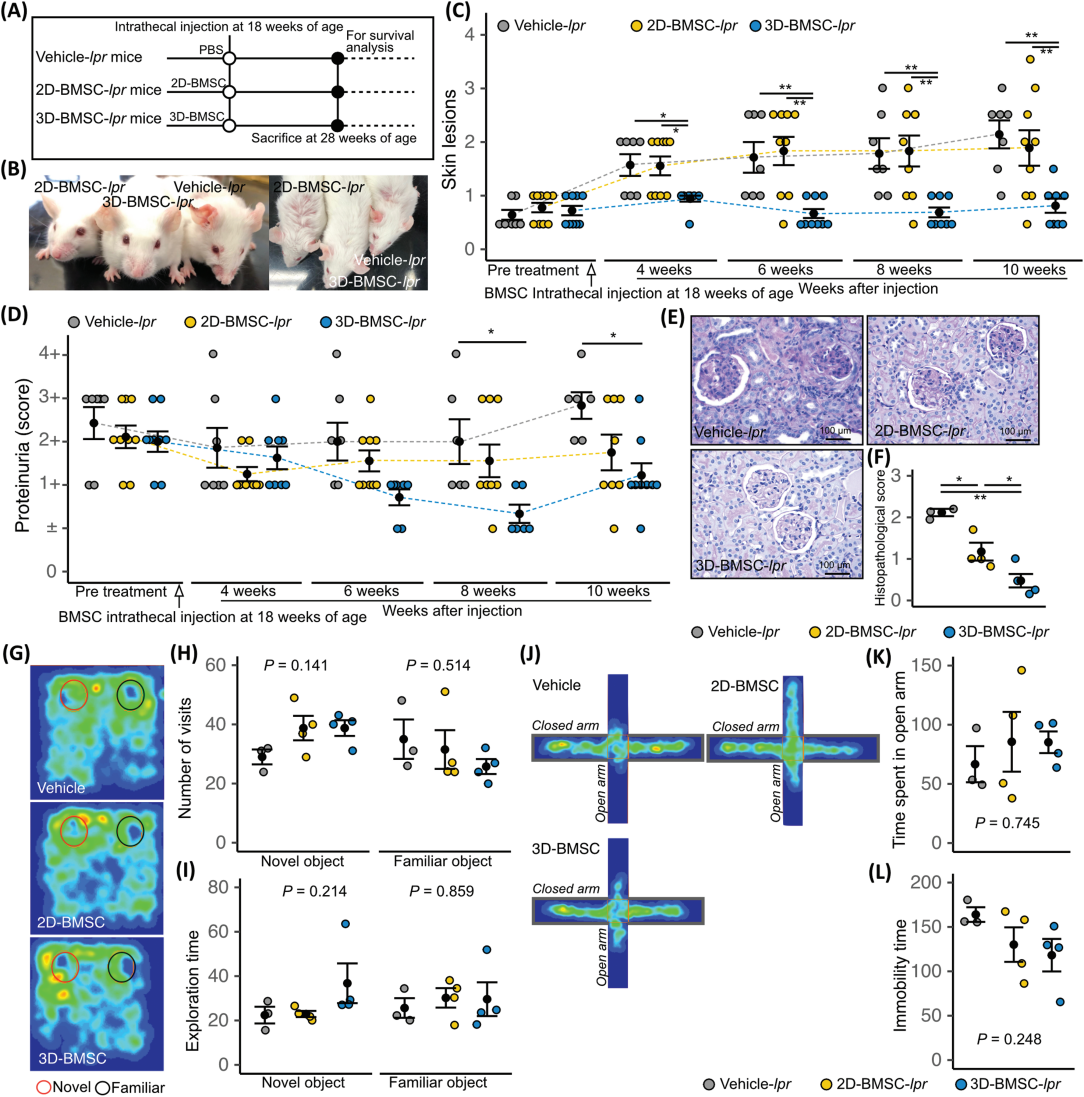
Intrathecal 3D-BMSC injection improves lupus-related symptoms. (**A**) Schematic diagram of the procedures for intrathecal MSC or 3D-BMSC injection in MRL/*lpr* mice. Representative images of skin lesions in vehicle-, MSC-, and 3D-BMSC–treated mice (**B**), and the quantitative data (**C**). (**D**) Semi-quantitative data of proteinuria after MSC or 3D-BMSC treatment. Representative images of PAS staining of kidneys from vehicle-, MSC-, and 3D-BMSC–treated mice (**E**), and the quantitative data of histopathological scores (**F**). (**G**) Cumulative heat maps of the time spent during the object recognition test. A longer time spent is depicted in red and no time spent is depicted in black. Quantified data of the number of visits to the novel and familiar objects (**H**) and the time spent exploring each (**I**). (**J**) Cumulative heat maps of the time spent in the elevated plus-maze. A longer time spent is depicted in red and no time spent is depicted in black. (**K**) Quantified data of the time spent in the open arm. (**L**) Immobility time during tail suspension test. Quantitative data are shown as means ± SEs in dot plots. *P*-values were determined by one-way ANOVA adjusted by the Holm method (*P < .05, **P < .001).

**Figure 7.**
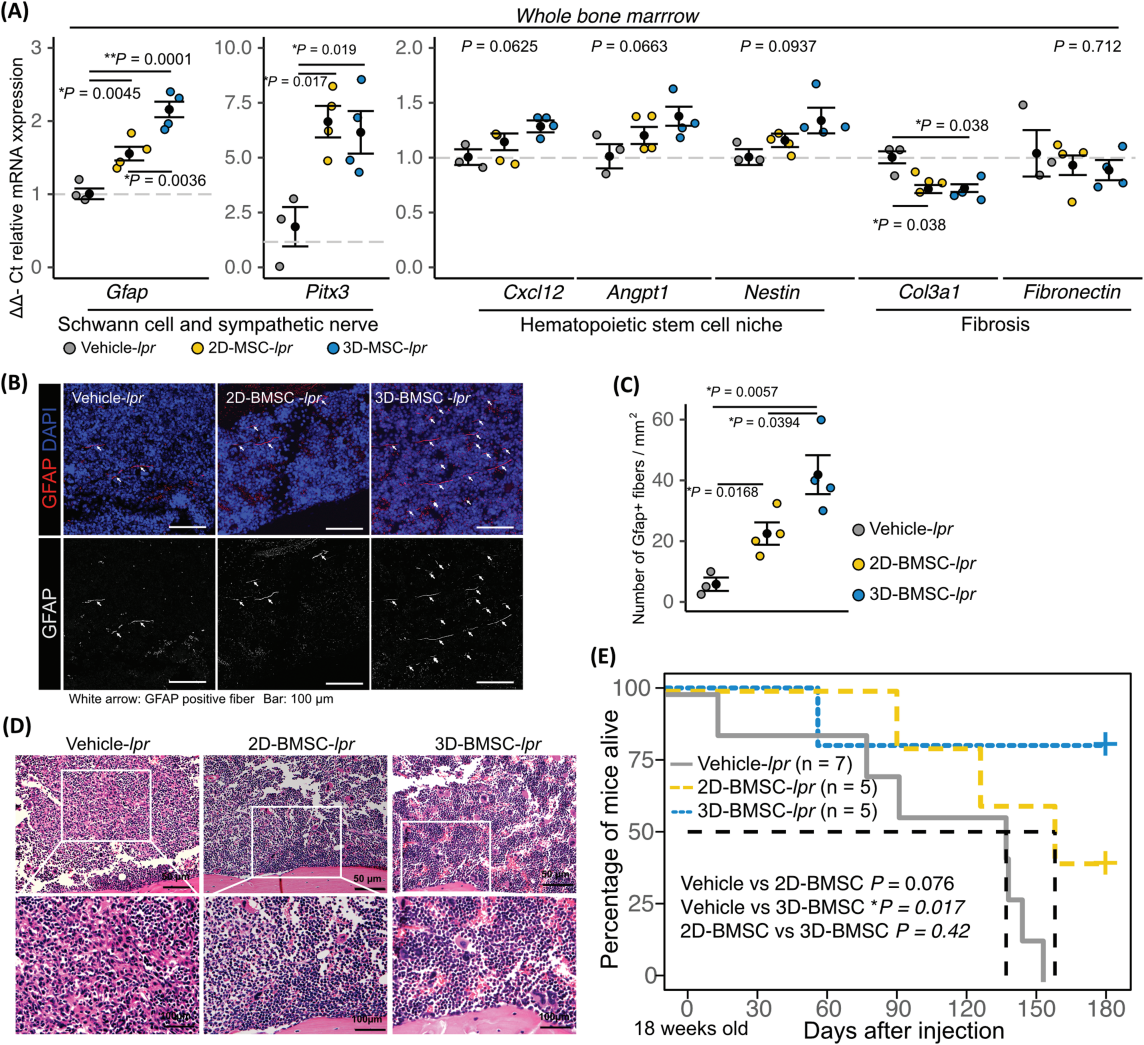
Intrathecal 3D-BMSC injection ameliorates bone marrow neuropathy and reduces mortality in lupus-prone mice. (**A**) Relative mRNA expression of genes related to Schwann cells, sympathetic nerves, the hematopoietic stem cells niche, and fibrosis in bone marrow after BMSC treatment. Representative images of GFAP immunostaining of bone marrow after BMSC treatment. Arrow indicates GFAP fiber (**B**). The quantified data of the number of GFAP positive fiber. (**C**, **D**) Representative image of HE staining of bone marrow after BMSC treatment. (**E**) Kaplan-Meier survival curves for vehicle-, BMSC-, and 3D-MSC–treated mice. Quantitative data are shown as means ± SEs in dot plots. *P*-values were determined by one-way ANOVA adjusted by the Holm method (*P < .05, **P < .001).

To investigate whether 3D-BMSCs had better therapeutic efficacy than 2D-BMSC, we performed lumbar intrathecal injections of 2D-BMSCs or 3D-BMSCs isolated from SLE patient, and PBS as vehicle in 18-week-old MRL/*lpr* mice (resulting in 2D-BMSC-*lpr* mice, 3D-BMSC-*lpr* mice, and Vehicle-*lpr* mice, respectively) (Fig. 6A). The 3D-BMSC-*lpr* mice demonstrated significant improvements in skin lesions, kidney histopathological score, and proteinuria (Fig. 6B-F), but not in cognitive defects, anxiety, or depression-like phenotype (Fig. 6G-L). The mRNA expression of *Pitx3* and *Gfap* and the number of GFAP-positive fibers were significantly increased in 3D-BMSC-*lpr* mice (Fig. 7A and B), whereas HSC niche factors were not (Fig. 7A). Bone marrow fibrosis was ameliorated and *Col3a1* mRNA expression was reduced in both BMSC-*lpr* mice and 3D-BMSC-*lpr* mice (Fig. 7A and D). We found the mRNA expression level of Schwann cell and sympathetic nerve and HSC niche factors have negative correlation with kidney histopathological score, proteinurea, skin lesions score, and tail suspension test, and have positive correlation with object recognition test and elevated plus-maze test, that mean improvement of kidney function, skin lesions, cognitive defect, anxiety and depression-like symptoms may potentially affected by the improved mRNA expression level of Schwann cell and sympathetic nerve and HSC niche factors by 3D-BMSC therapy (Supplementary Fig. S4A). In addition, we performed in vivo tracking of PKH26-labeled BMSC, and we could not detect PKH26-labeled BMSC in brain (Supplementary Fig. S4B). Finally, Kaplan-Meier survival curves showed a significant improvement in the survival rate of 3D-BMSC-*lpr* mice (Fig. 7E). We also tested whether control BMSC, which is isolated from no history of autoimmune disease individuals, exhibit therapeutic effects to multiple organ damage in MRL/*lpr* mice, we performed lumbar intrathecal injections of 2D-BMSC and 3D-BMSC in 18-week-old MRL/*lpr* mice. The administration of 3D-BMSC isolated from no history of autoimmune disease individuals exhibits higher therapeutic effect than that of 2D-BMSC isolated from no history of autoimmune disease individuals in skin lesion, kidney histopathological score, and proteinuria (Supplementary Fig. S5A-E). In recognition memory test, the exploration time in vehicle mice significantly decreased in vehicle MRL/*lpr* mice but not significantly difference between MRL/*Mpj* mice and 2D-BMSC and 3D-BMSC treated MRL/*lpr* mice (Supplementary Fig. S5F-S5G). These results suggest that the intrathecal injection of 3D-BMSC is therapeutically effective for improving bone marrow neuropathy and SLE-related symptoms.

## Discussion

CNS inflammation is caused by autoreactive lymphocytes, as in multiple sclerosis (MS) pathogenesis,^33,34^ and experimental autoimmune encephalomyelitis (EAE) model mice exhibit locus ceruleus damage and reduction in the level of noradrenaline.^35^ In MRL/*lpr* mice, TH-positive dopaminergic neurons were less abundant, and GFAP-positive cells were more abundant, in locus ceruleus of mice, the major source of noradrenaline.^21^ Patients with SLE frequently exhibit CNS-involved symptoms, termed neuropsychiatric SLE (NPSLE), including nonspecific symptoms such as headache, cognitive abnormalities, and mood disorder; in addition, they present with peripheral nervous system impairments such as polyneuropathy and autonomic dysfunction.^4,36,37^ Bone marrow is a peripheral organ with direct sympathetic innervation,^7^ and bone marrow abnormalities have been reported in patients with SLE.^10,11^ Hence, in this study, we focused on bone marrow neuropathy. The functional importance of bone marrow innervation is well studied in the context of the HSC niche.^8^ SNS in bone marrow regulates the mobilization of hematopoietic stem and progenitor cells and hematopoietic regeneration following genotoxic stress,^8,25,38^ and GFAP-positive nonmyelin Schwann cells also contribute to the HSC niche by promoting HSC quiescence.^39^ We showed that MRL/*lpr* mice exhibit bone marrow neuropathy, as demonstrated by reduced expression of *Gfap* and *Pitx3* and reductions in GFAP-positive fibers in bone marrow, which might induce excessive HSC expansion and mobilization of bone marrow cells into peripheral organ, resulting in multiple organ damage.

To better understand the relationship between SNS damage and bone marrow neuropathy with SLE symptoms, we tested the effect of the neuroprotective drug 4MC and the sympathetic neurotoxic drug 6OHDA. Arranz and colleagues reported that the 4MC protects the SNS, as well as Schwann cells, which function as stem cell niches to prevent mutant HSC expansion, thereby averting bone marrow fibrosis and cytokine storm.^9^ They also reported that by protecting against bone marrow neuropathy, 4MC prevents excessive expansion of circulating neutrophils.^9^ Neutrophils contribute to the pathogenesis of lupus nephritis.^40,41^ The effects of and 4MC are not likely to be bone marrow specific, but we speculated that alleviation of bone marrow neuropathy by 4MC treatment could underlie the amelioration of renal impairment. 6OHDA is known to induce SNS fibers defects in bone marrow, which affects the HSC microenvironment.^25,38,42^ del Rey et al reported that sympathetic denervation by 6OHDA treatment increased the concentration of IgM and IgG2a in plasma and shortened the survival time in MRL/*lpr* mice than in control mice.^43^ Our study demonstrated that 6OHDA treated-MRL/*lpr* mice exhibited glomerulonephritis and cell infiltration into the tubulointerstitial space, proteinuria, and skin lesions. These results suggest that bone marrow neuropathy can promote progression of multiple-organ damage in lupus model mice. Further studies are needed to reveal additional mechanisms of bone marrow neuropathy-mediated multiple-organ damage in lupus.

To develop SLE treatment aimed at regenerating the nervous system, we focused on BMSC therapy because BMSCs have neuro-protective and neuro-regenerative effects.^27^ In humans, the safety and therapeutic effect of intrathecal BMSC administration have been reported in amyotrophic lateral sclerosis, spinal cord injury, and multiple sclerosis.^26^ Our results showed that the administration of BMSCs derived from patients with SLE did not improve renal function or mortality. Since patients with SLE develop bone marrow fibrosis and myelodysplastic syndromes,^10^ we hypothesized that BMSCs derived from the bone marrow of patients with SLE would show a decreased therapeutic effect, while re-activation of these BMSCs would increase it. In this study, we used a 3D fiber scaffold whose micro/nanoscale topography causes changes in the cell morphology and stem cell fate of MSCs, thus improving these cells’ therapeutic effect.^44^ We found that culturing 3D-BMSCs on this scaffold upregulated the mRNA expression of *POU5F1*, *IDO1*, and *GDNF* and downregulated that of *ACTA2*. While the detailed mechanism underlying BMSC therapy is still unknown, it was reported that the upregulation of *POU5F1* and *IDO1* and the downregulation of *ACTA2* served as biomarkers for therapeutically effective BMSCs.^45,46^ MSC with enhanced GDNF expression strongly improved the therapeutic effect on diseases such as ALS in vivo,^47-51^ and that GDNF itself has neuroprotective effects.^31,52-55^ It was shown that intrathecal injection of BMSCs that enhanced GDNF expression showed therapeutic effects in patients with amyotrophic lateral sclerosis in phase I/II and 2a clinical trials.^51^ This study showed that 3D-BMSC increased the *GDNF* expression, and promotes sympathetic nerve differentiation of TGW human neuroblastoma by coculture experiments, and decreases TH expression by GDNF antibody, which indicates GDNF secrete from 3D-BMSC affect to the TH expression in TGW cells. In addition, CM isolated from 3D-BMSC promote sympathetic nerve differentiation of TGW cells than that of CM of 2D-BMSC. The difference of cell size between 2D-BMSC and 3D-BMSC may also indicate the difference of the therapeutic effect. In our present study, we showed several distinctive features of 3D-BMSC that is smaller size than that of 2D-BMSC. It has been widely reported that cell size and morphology affects the therapeutic effect of MSC.^56-59^ These previous studies showed the features of small size MSC that are high expression of NANOG, POU5F1 and IDO1, and lower expression of α-SMA.^58,59^ Further, small size MSC exhibit higher therapeutic effect on elastase-induced emphysema model and myosin-induced experimental autoimmune myositis by inhibiting chronic inflammation.^46,58^ These results indicated that multi factors including GDNF improved by 3D-BMSC, which enhanced the therapeutic effect of BMSC to MRL/*lpr* mice.

Although 3D-BMSC exhibits a beneficial effect on skin lesions, renal function, and mortality, the effect on cognitive defect and anxiety was limited. Our results also showed that PKH26-labeled BMSC could not identify in the brain. In previous studies which reported the distribution of intrathecal injected MSCs, injected MSC migrated to the brain around 12-hour, and few MSCs remained after 24-hour.^60^ They also showed the efficacy of MSC migration to the brain was depend on the number of injected MSCs.^60^ Another study reported that intracerebroventricularly (ICV) injection of MSCs distributed lateral ventricles at 7 days after injection, but few MSCs were distributed at 21 days after injection.^61^ In a systematic review by Sanchez-Diaz and their colleagues in 2021, ICV injection of MSC was detected at 1-3 weeks after injection and then, decreased time-dependently.^62^ Another study showed that MSCs can be detected in the brain up to 7 weeks after ICV injection.^63^ The efficacy of MSC distribution in the brain may rely on the distance between injected site and brain, and the number of injected MSCs, hence, intrathecal injection at L4 and L5 levels may have a lower effect on the brain in this study. This study performed that the intrathecal injection at L4 and L5 for the treatment of bone marrow neuropathy. SNS represents the major arm of the CNS. Preganglionic neurons from the thoracolumbar spinal cord from sympathetic ganglia from which postganglionic neurons innervate various organs including bone marrow.^28^ It is possible that the anatomical location of intrathecal injection at the lumbar site has increased its effectiveness against bone marrow neuropathy. Although we could not find the therapeutic effect of neuropsychiatric SLE symptoms, additional statistical analysis showed that the mRNA expression level of Schwann cell and sympathetic nerve and HSC niche factors have a positive correlation with object recognition test and elevated plus-maze test and a negative correlation with tail suspension test. Intrathecal injection of 3D-BMSC may potentially affect the brain by indirect effect via improvement of bone marrow neuropathy or by direct migration of a few injected MSCs to the brain transiently. Further study will be needed to justify the injected site or routes to affect not only bone marrow neuropathy but also the brain.

In this study, we used human BMSCs in anticipation of future clinical applications. The effect of (and response to) xenotransplantation of BMSCs may differ from that of transplantation of allogenic or autogenic BMSCs.^45^ However, Galleu et al reported that the results of treating GvHD patients with human mesenchymal stromal cells (MSCs) were similar to those of treating a GvHD mouse model with human MSCs, and that mouse inflammatory cytokines did not activate immunomodulatory in human MSCs.^64^ Therefore, in order to evaluate the therapeutic potential of human MSCs for future clinical therapeutic applications, we performed this study using human MSCs. Regarding the cell source, we used bone marrow from patients with SLE and non- patients with SLE (osteoarthritis) who performed total hip replacement. Although this additional experiment was conducted by non-patients with SLE-derived BMSC because of the limited availability of patients with SLE-derived cells, the therapeutic difference between 2D-BMSC and 3D-BMSC was found in both SLE- and non-SLE–derived cells (Supplementary Fig. S5) in vivo. It is speculated that SLE-derived 3D-BMSC-CM may increase the TH expression when compared with SLE-derived 2D-BMSC-CM like non-SLE patient-derived cells.

Several studies have investigated MSC therapy in MRL/*lpr* mice. The BMSCs in these studies were commonly derived from human or mouse bone marrow, adipose tissue, placenta, and umbilical cord, and when administered intravenously they reduced inflammation via T-cell regulation and ameliorated renal dysfunction.^65-69^ Gu et al reported that BMSCs from the bone marrow of patients with SLE showed only a slightly improved effect on the survival rate of MRL/*lpr* mice compared with treatment with BMSCs from the bone marrow of healthy humans, but the effect was improved by inhibiting the mTOR pathway with rapamycin before cell administration.^65^ Similarly, we found that the survival rate of MRL/*lpr* mice was improved when BMSCs derived from patients with SLE were pre-conditioned by culture on a 3D fiber scaffold. In long-term survival analyses of MRL/*lpr* lupus-prone mice, cyclophosphamide treatment improved survival by 22.2% at 300 days of age compared with vehicle,^16^ and Ras-related in brain 7 (Rab7) inhibition by CID 1067700 improved survival by 62.5% at 300 days of age compared with vehicle.^70^ In our study, intrathecal 3D-BMSC administration improved survival by 80% at 300 days of age compared with vehicle; therefore, our approach has the potential to be another therapeutic strategy for SLE.

## Conclusion

Our study highlights the contribution of bone marrow neuropathy and multiple organ damage in a murine lupus model. Neuroprotective effects of intrathecal BMSC injection may provide another therapeutic option for restoring bone marrow and alleviating multiple organ damage. In addition, BMSCs had a decreased therapeutic effect when derived from patients with SLE; therefore, we propose pre-conditioning these cells by culturing them on 3D fiber scaffolds to improve the benefits of autologous BMSC therapy.

## Supplementary Material

szac021_suppl_Supplementary_Material

## Data Availability

Data and materials not indicated in this manuscript are available from the corresponding author.
