# Supplementary material for: Intrathecal Injection of Mesenchymal Stromal Cell Cultured on 3D Fiber Ameliorates Multiple Organ Damage in Murine Lupus
Source: Stem Cells Transl Med. 2022 Apr 25;11(6):644–58. doi: 10.1093/stcltm/szac021 (PMC9216504; doi:10.1093/stcltm/szac021)
Supplement: szac021_suppl_Supplementary_Material [file szac021_suppl_supplementary_material.pdf]

Supplementary File for

Intrathecal injection of mesenchymal stromal cells cultured on 3D fiber ameliorates multiple organ damage in murine lupus

Authors: Yuki Saito PhD1, Maki Miyajima PhD2, Sena Yamamoto2, Norihiro Miura2, Tsukasa Sato2, Arisa Kita MD, Shogo Ijima D.D.S4, Mineko Fujimiya MD, PhD1, Takako S. Chikenji PhD 1,2*

Affiliations:

1 Department of Anatomy, Sapporo Medical University School of Medicine, Sapporo, Japan.
2 Graduate School of Health Sciences, Hokkaido University, Sapporo, Japan.
3 Department of Plastic and Reconstructive Surgery, Sapporo Medical University, Sapporo, Japan.
4 Department of Oral Surgery, Sapporo Medical University School of Medicine, Sapporo, Japan.

***Corresponding author: Takako S. Chikenji.**

Address: North12 West5, Kitaku, Sapporo, 060-0812, Japan
Telephone: 011-706-3382 FAX: 011-706-3382
Email: chikenji@pop.med.hokudai.ac.jp

Supplementary Figures

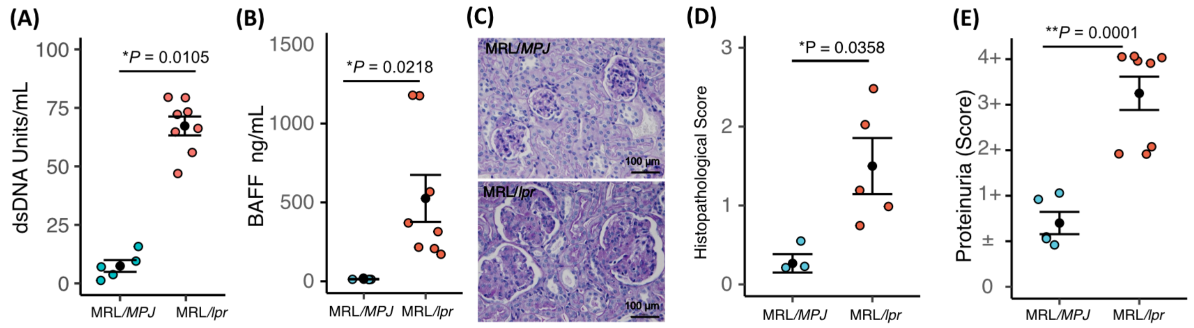

Supplementary Figure 1. SLE symptoms in MRL/*lpr* mice. (A) Serum concentration of anti-dsDNA antibodies and (B) B cell–activating factor (BAFF) in MRL/*MPJ* and MRL/*lpr* mice. (C) Representative images of PAS staining of kidneys from MRL/*MPJ* (n = 3) and MRL/*lpr* mice (n = 5). (D) Semi-quantitative data of proteinuria in MRL/*MPJ* (n = 3) and MRL/*lpr* mice (n = 5). Quantitative data are shown as means ± SEs in dot plots. P-values were determined by two-

tailed Student's t-test. (*P < 0.05, **P < 0.001)

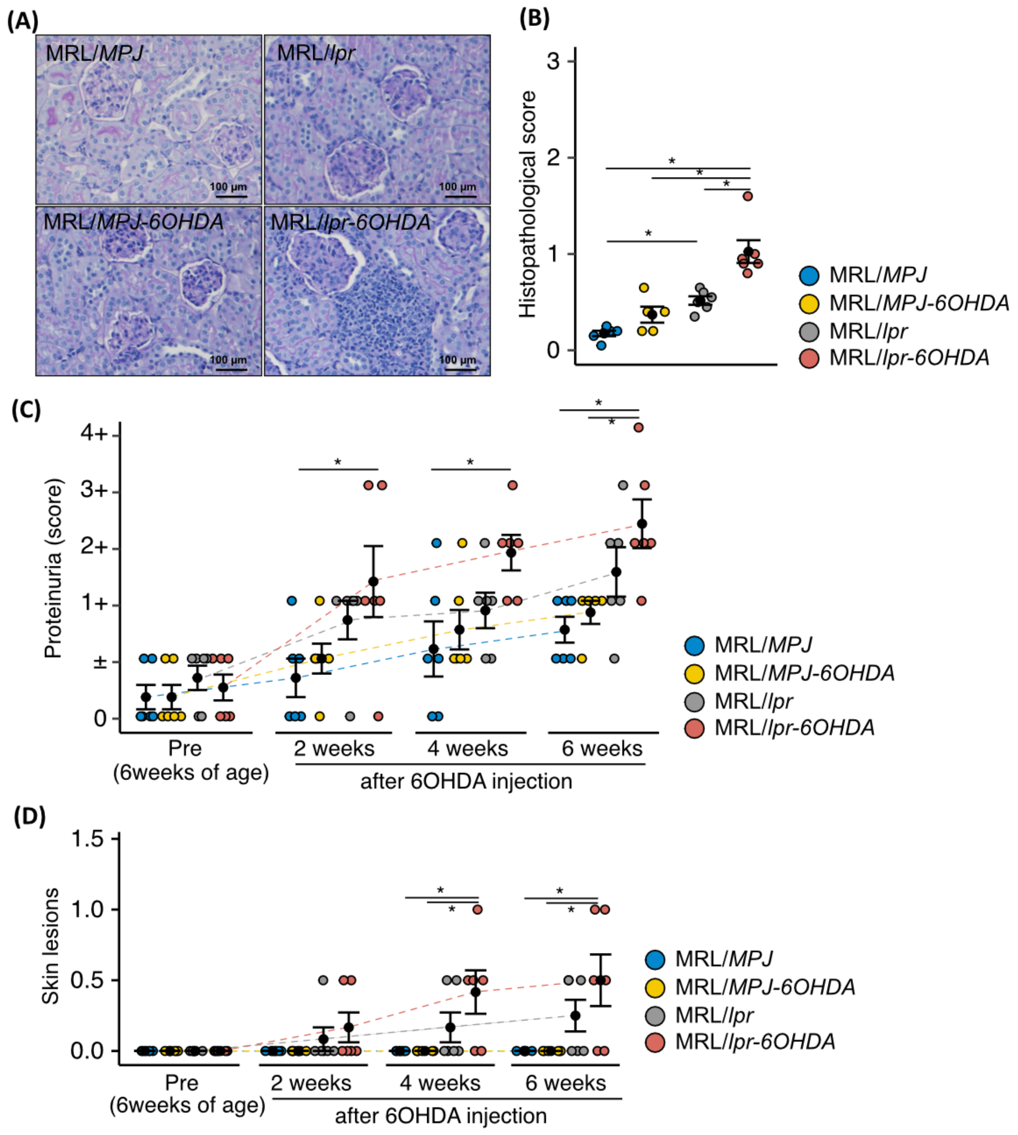

Supplementary Figure 2. *6OHDA administration induces multiple organ damage in lupus-prone mice but not in non–lupus-prone mice.* (A–B) Representative images of PAS staining of kidneys from MRL/*Mpj* and MRL/*lpr* mice with or without 6OHDA administration (A), and quantitative data of histopathological scores (B). (C–D) Semi-quantitative data of proteinuria (C) and quantitative data of skin lesions (D) after 6OHDA administration to MRL/*lpr* mice. Quantitative data for each specimen are shown as means ± SEs in dot plots. P-values were determined by two-tailed Student's t-test or one-way ANOVA adjusted by the Holm method. (*P < 0.05)

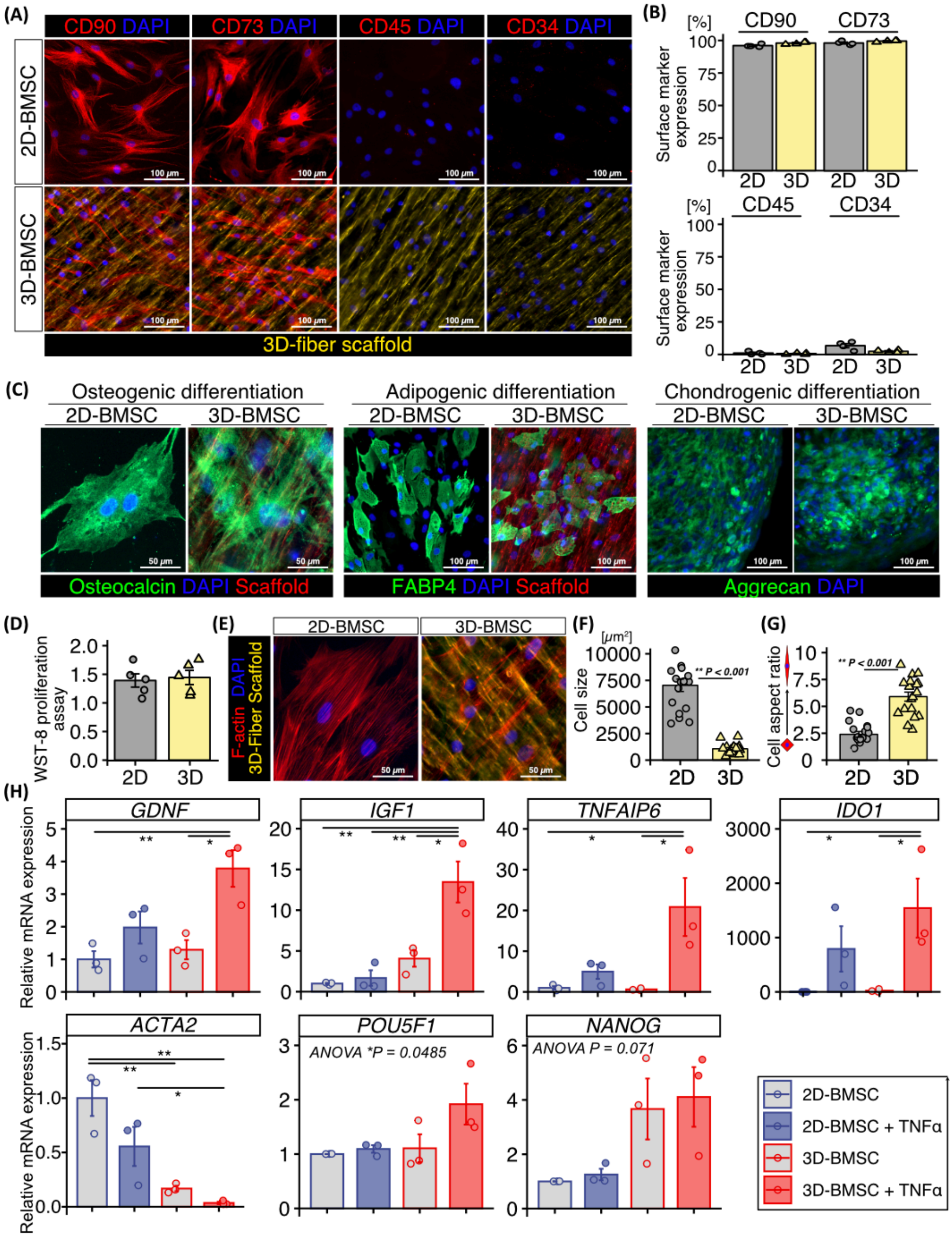

Supplementary Figure ***3. Characteristics of 2D- and 3D-BMSCs.***

(A–B) Representative images of CD90, CD73, CD45, and CD34 immunostaining of 2D- and 3D-BMSCs (A), and the quantitative data (n =4) (B). (C) Representative images of osteocalcin, FABP4, and aggrecan immunostaining of 2D- and 3D-BMSCs after osteogenic, adipogenic, and chondrogenic differentiation, respectively. (E–G) Representative images of F-actin staining of 2D- and 3D-BMSCs (E), and quantitative data of cell size (F) and cell aspect ratio (G). Relative mRNA expression of *GDNF*, *IGF1*, *TNFAIP6*,

IDO1, *ACTA2*, *POU5F1*, and *NANOG* in 2D- and 3D-BMSCs after TNF stimulation. Quantitative data for each specimen are shown as means ± SEs in dot plots. P-values were determined by two-tailed Student's t-test or one-way ANOVA adjusted by the Holm method. (*P < 0.05, **P < 0.001)

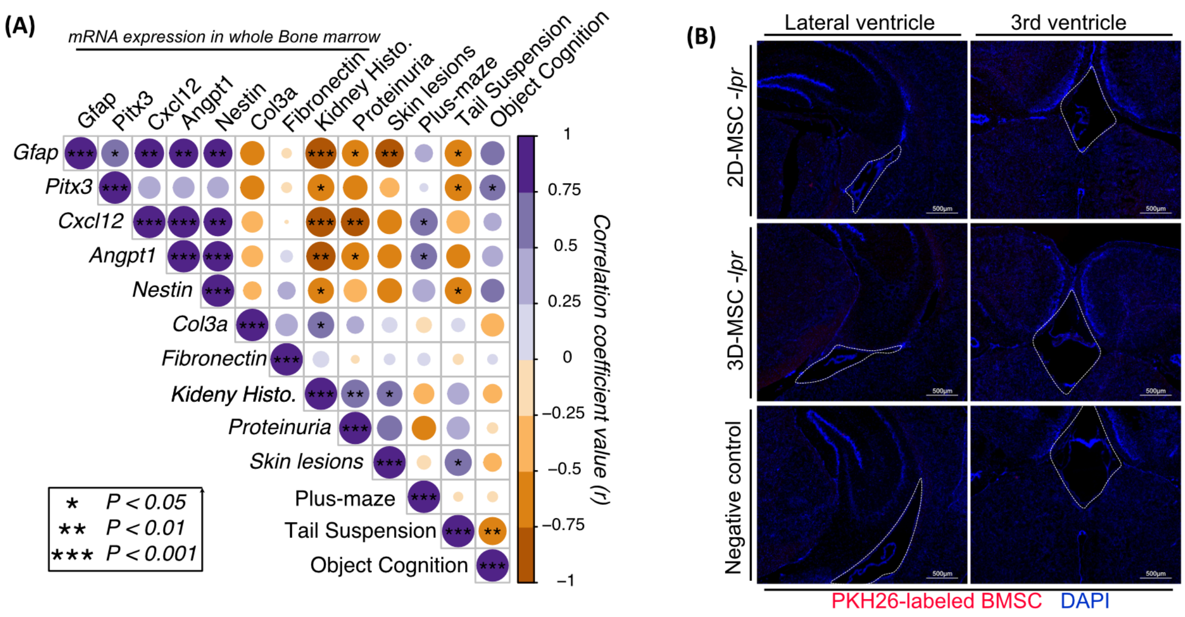

Supplementary Figure ***4. Correlation plot of bone marrow mRNA expression and kidney, skin, and behavioral test outcomes, and the distribution of intrathecally injected BMSCs.***

(A) Correlation plot of the expression of mRNA related to Schwann cells, sympathetic nerves, the HSC niche factors, kidney histopathological scores, proteinuria, skin lesion scores, and behavioral tests. Positive correlations are depicted in purple, and negative correlations are depicted in orange. (B) Localization of PKH-labeled 2D- and 3D-BMSCs in the brain.

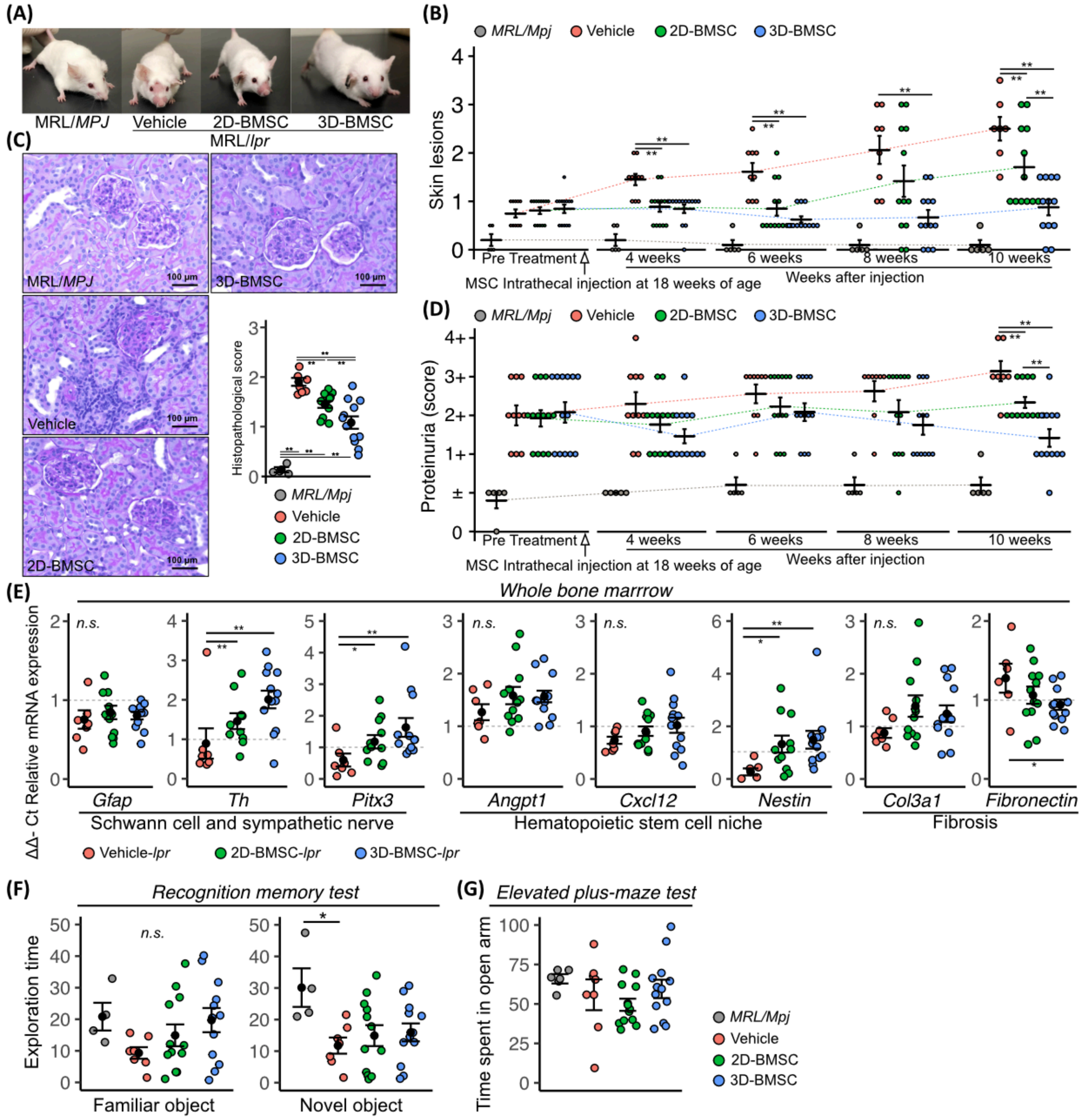

Supplementary Figure 5. ***Lupus-prone mice injected intrathecally with BMSCs derived from patients without autoimmune disease.***

(A–B) Representative images of skin lesions in MRL/*MPJ* mice and vehicle-, 2D-BMSC–, and 3D-BMSC–treated MRL/*lpr* mice (A), and the quantitative data (B). (C) Representative images of PAS staining of kidneys from MRL/*MPJ* mice and vehicle-, 2D-BMSC–, and 3D-BMSC–treated MRL/*lpr* mice, and the quantitative data of the histopathological scores. (D) Semi-quantitative data of proteinuria after BMSC treatment. (E) Relative mRNA expression of genes related to Schwann cells, sympathetic nerves, the HSC niche, and fibrosis in bone marrow after BMSC treatment. (F) Quantitative data of the exploration

times of novel and familiar objects during the object recognition test. (G) Quantitative data of the time spent in the open arm during the elevated plus-maze. Quantitative data for each specimen are shown as means ± SEs in dot plots. P-values were determined by two-tailed Student's t-test or one-way ANOVA adjusted by the Holm method. (*$P < 0.05$, **$P < 0.001$)

Supplementary table 1. Specific primer sequences used for real-time PCR

Gene	Forward	Reverse	Size	Accession Number
Mouse				
Gfap	5' CGGAGACGCATCACCTCTG 3'	5' AGGGAGTGGAGGAGTCATTCG 3'	126	NM_001131020.1
Pitx3	5' GACACTGGCCGCCCAAGG 3'	5' AGGCCCCACGTTGACCGA 3'	83	NM_008852.4
Cxcl12	5' TGCATCAGTGACGGTAAACCA 3'	5' TTCTTCAGCCGTGCAACAATC 3'	146	NM_021704.3
Angpt12	5' CTCGTCAGACATTCATCATCCA 3'	5' CACCTTCTTTAGTGCAAAGGCT 3'	138	NM_009640.4
Nestin	5' CCCTGAAGTCGAGGAGCTG 3'	5' CTGCTGCACCTCTAAGCGA 3'	166	NM_016701.3
Col3a1	5' TGGAGGATGGTTGCACGAAA 3'	5' ACAGCCTTGCGTGTTCGATA 3'	73	NM_009930.2
Fibronectin	5' ATCTGGACCCCTCCTGATAGT 3'	5' GCCCAGTGATTTCAGCAAAGG 3'	124	NM_010233.2
Gapdh	5' AGGTCGGTGTGAACGGATTTG 3'	5' TGTAGACCATGTAGTTGAGGTCA 3'	123	NM_001289726.1
Actb	5' CATTGCTGACAGGATGCAGAAGG 3'	5' TGCTGGAAGGTGGACAGTGAGG 3'	138	NM_007393.5
Human				
POU5F1	5' GACAGGGGAGGGGAGGAG 3'	5' CTTCCCTCCAACCAGTTGCCC 3'	143	NM_001159542
NANOG	5' TGGACACTGGCTGAATCCTTC 3'	5' CGTTGATTAGGCTCCAACCAT 3'	142	NM_024865.4
IDO1	5' GCATTTTTCAGTGTTCTTCGCATA 3'	5' TCATACACCAGACCGTCTGATAGC 3'	77	NM_002164
TNFAIP6	5' CCCATTGTGAAGCCAGGGCCCAACTG 3'	5' GGAAGCTCATCTCCACAGTATCTTCCC 3'	362	NM_007115
ACTA2	5' GCAGCCCAGCCAAGCACTGT 3'	5' TGGGAGCATCGTCCCCAGCA 3'	135	NM_001613
GDNF	5' CGCCGAAGACCGCTCCCTCG 3'	5' ATCCATGACATCATCGAACTGATC 3'	103	NM_000514.4
IGF-1	5' CTCTTCAGTTCGTGTGTGGAGAC 3'	5' CAGCCTCCTTAGATCACAGCTC 3'	134	NM_001111283.3
ACTB	5' AGAGCTACGAGCTGCCTGAC 3'	5' AGCACTGTGTTGGCGTACAG 3'	184	NM_001101.5
GAPDH	5' ATTGCCCTCAACGACCACTT 3'	5' TGCTGTAGCCAAATTCGTTGTC 3'	64	NM_002046
